# Comparison of Microbial Populations in Saliva and Feces from Healthy and Celiac Adolescents with Conventional and Molecular Approaches after Cultivation on Gluten-Containing Media: An Exploratory Study

**DOI:** 10.3390/microorganisms9112375

**Published:** 2021-11-17

**Authors:** Tilen Senicar, Andraz Kukovicic, Valerija Tkalec, Aleksander Mahnic, Jernej Dolinsek, Maja Rupnik

**Affiliations:** 1 Faculty of Medicine, University of Maribor, 2000 Maribor, Slovenia; tilen.senicar@gmail.com (T.S.); andy.kukovicic@gmail.com (A.K.); valerija.tkalec@nlzoh.si (V.T.); aleksander.mahnic@nlzoh.si (A.M.); 2 National Laboratory for Health, Environment and Food, 2000 Maribor, Slovenia; 3 Department of Paediatrics, University Clinical Centre Maribor, 2000 Maribor, Slovenia; Jernej.DOLINSEK@ukc-mb.si

**Keywords:** gluten-degrading microorganisms, *Veillonella*, *Candida*, short-chain fatty acids, celiac disease, microbiota, sequencing

## Abstract

Microbes capable of metabolizing gluten are common in various parts of the intestinal tract. In this study, saliva and fecal samples were obtained from 10 adolescents (13–18 years of age), five of which had celiac disease (CD) and five of which were healthy volunteers (HV). Culture-enriched saliva and fecal samples were compared with molecular profiling, and microorganisms displaying lysis zones on gluten-containing media (i.e., gluten-degrading microorganisms; GDMs) were isolated. In total, 45 gluten-degrading strains were isolated, belonging to 13 genera and 15 species, including *Candida albicans* and *Veillonella*. GDMs were more common in HVs compared to CD patients and more diverse in saliva compared to feces. In saliva, GDMs showed partial overlap between HVs and CD patients. Bacterial communities in fecal samples determined with amplicon sequencing significantly differed between CD patients and HVs. Overall, 7–46 of all operational taxonomic units (OTUs) per sample were below the detection limit in the fecal samples but were present in the cultivated samples, and mainly included representatives from *Lactobacillus* and *Enterococcus*. Furthermore, differences in fecal short-chain fatty-acid concentrations between CD patients and HVs, as well as their correlations with bacterial taxa, were demonstrated.

## 1. Introduction

Celiac disease (CD) is a chronic inflammatory disease with a global prevalence of approximately 1%. Inflammation in CD occurs due to abnormal immune responses after gluten intake in genetically susceptible individuals [1]. Microbiota from different intestinal tract niches and their metabolites can be associated with CD in several ways [2,3,4,5,6]. Salivary and fecal microbiota differ between CD patients and healthy controls [2,3,4,7]; however, no disease-specific microbial signatures have been described. Nevertheless, specific changes were associated with different clinical manifestations of the disease [2]. In a murine model, microbiota either exhibited a protective role or exacerbated the immune response to gluten, depending on the bacterial community composition [2,8]. Lastly, microbiota can serve as a reservoir of enzymes for gluten degradation [8], which can result in products with either increased or decreased immunogenic properties [2,9,10].

Various gluten-degrading microbial species (GDMs) have been previously isolated, predominantly from saliva and feces, due to the ease of sampling [11,12,13,14]. Among the first isolated microorganisms capable of degrading various gluten components were *Rothia mucilaginosa* and *Rothia aeria* [12,15]. Many representatives of the genus *Lactobacillus* can also degrade gluten and are more often found in the oral cavity of healthy individuals than in that of CD patients [16]. To a lesser extent, the ability to break down gluten has also been demonstrated in various *Streptococcus* species, which, despite their lower enzymatic activity, play an important role in gluten breakdown due to their abundance [13,17].

Caminero and colleagues have provided the most extensive studies regarding microorganisms capable of metabolizing gluten [10,16,18]. Of the 144 isolated strains, 73% belonged to the phylum Firmicutes, 15% belonged to the phylum Actinobacteria, and 12% were Gram-negative bacteria from the phylum Proteobacteria. Extracellular proteolytic activity against immunogenic gluten peptide sequences was demonstrated in 61 of the isolated strains [18].

The aim of this study was to compare microbial populations from the saliva and feces of adolescent healthy volunteers (HVs) and CD patients by combining different cultivation approaches and sequencing. GDMs were isolated on gluten-containing media. Different cultivation approaches (e.g., direct plating and enrichment) were used to broaden the spectrum of captured GDMs. Additionally, fecal bacterial community structure and short-chain fatty acid (SCFA) profiles were compared between CD patients and HVs.

## 2. Materials and Methods

### 2.1. Sampling

Fecal and saliva samples were obtained from five CD patients on a gluten-free diet (two female, three male) and five HVs (three female, two male). The participants were between 13 and 18 years old. The CD patients were recruited at the Department of Pediatrics, University Clinical Centre Maribor. Informed consent forms were obtained for all 10 participants at the beginning of the study. Ethical approval was obtained from the Ethics Committee of the University Medical Centre Maribor (UKC–MB–KME–72/20).

Fecal samples were collected in sterile containers and stored at 4 °C. Saliva samples were collected in the morning before the participants brushed their teeth. The participants were asked to rinse their mouth with water two times, allow saliva to pool in their mouth for approximately 5–10 min, and collect their saliva (approximately 2 mL) into a 20 mL sterile tube. All collected samples were received in the laboratory within 24 h of collection, stored at 4–8 °C, and processed within 24 h.

### 2.2. Isolation of GDMs

MCG3 broth and agar medium with gluten as the main source of nitrogen were prepared as described by Caminero et al. [18]. Each sample was cultivated by four parallel approaches: direct plating under aerobic (1) or anaerobic (2) conditions or initial enrichment followed by plating under aerobic (3) or anaerobic (4) conditions. For anaerobic cultivation, all media were pre-reduced in an anaerobic atmosphere (80% N_2_, 10% CO_2_, 10% H_2_; Anaerobic Workstation, Don Whitley Scientific, Bingley, UK) for at least 24 h, and then the entire experiment was set up in the anaerobic atmosphere.

For fecal sample analysis, approximately 0.5 g of fecal sample was suspended in 3 mL of saline (0.85% NaCl). For enrichment, the fecal suspension (0.5 mL) and undiluted saliva samples (0.5 mL) were inoculated into 5 mL of MCG3 broth and incubated for 72 h at 37 °C. Subsequently, 1 mL of enrichment culture was centrifuged, and the pellet was stored in 1 mL of Inhibitex buffer (QIAGEN, Düsseldorf, Germany) at −80 °C for 16S rRNA amplicon sequencing. Additionally, enrichment culture dilutions (10^−2^ to 10^−4^) were plated onto MCG3 agar and incubated for 7 days at 37 °C.

For direct sample cultivation, tenfold serial dilutions of fecal suspensions or saliva (from 10^−2^ to 10^−6^) were prepared in saline, plated onto MCG3 agar plates, and incubated for 7 days at 37 °C. Isolates with observed lysis zones were subcultured, and pure cultures were stored at −80 °C (Microbank, Pro-Lab diagnostics, Wirral, UK). All obtained isolates were identified using the MALDI Biotyper (Bruker Daltonics, Bremen, Germany).

### 2.3. Bacterial 16S RNA Metagenome Sequencing of Fecal Samples and Cultivated Fractions

After the individual colonies with lysis zones were picked, the entire plate was swabbed and resuspended in 1 mL of Inhibitex buffer (QIAGEN). For each individual sample and each cultivation approach (i.e., enriched/nonenriched, aerobic/anaerobic), swabs from all plated dilutions were pooled and stored at −80 °C for 16S rRNA amplicon sequencing.

From fecal samples and pellets of cultivated samples (saliva and feces), total DNA for 16S RNA sequencing was isolated using the QIAamp Fast DNA Stool Mini Kit (QIAGEN) according to the manufacturer’s instructions. Cells were lysed with SeptiFast Lys kit and MagNA Lyser (Roche Diagnostics, Mannheim, Germany). Sequencing of the bacterial 16S RNA metagenome (V3V4 variable region) was carried out as previously described [19]. Briefly, mothur (v.1.44.0) was used for quality filtering of sequence reads, as well as downstream analysis. Taxonomy was inferred using the RDP 16S rRNA reference base (Ribosomal Database Project, version 18). In total, we obtained 3,969,337 quality filtered reads, on average 30,533.4 per sample (SD = 11,475.3). We removed reads with an abundance of less than 0.001%, and each sample was subsampled to 10,000 reads.

Downstream statistics included analysis of alpha diversity (community richness and diversity) and beta diversity (AMOVA), all performed in mothur (v.1.44.0).

### 2.4. Quantification of Fecal SCFAs

To determine SCFA concentrations in the collected fecal samples, 25 mL of sterile, distilled water was added to 0.5 g of feces. The suspension was vortexed, shaken on a stirrer (at 1200 rpm for 20 min at room temperature), and centrifuged twice (at 10,000 rpm and 14,500 rpm for 10 min at room temperature). The supernatant was filtered through a 0.2 μm filter, aliquoted, and stored at −80 °C until analysis. The SCFA profiles were determined by the Department of Microbiology and Microbial Biotechnology, Biotechnical Faculty, University of Ljubljana. Immediately after thawing, sulfuric acid was added to reach pH 2. SCFAs were extracted with ether and analyzed with gas chromatography.

We compared the average SCFA concentrations between CD patients and HVs with the Student’s *t*-test, and *p* < 0.05 was considered significant.

## 3. Results

Saliva and fecal samples from adolescent HVs and CD patients were cultivated under aerobic or anaerobic conditions on gluten medium either directly or after enrichment. Additionally, the bacterial populations from the original samples (feces only) and various cultivation conditions were characterized by 16S rRNA amplicon sequencing, and SCFA concentrations were determined in fecal samples.

### 3.1. Bacterial Community Structure of Fecal Samples from CD Patients and HVs

The fecal bacterial communities significantly differed between CD patients and HVs (AMOVA, *p* = 0.04). Several representatives from the order Clostridiales were less abundant in CD patients compared to HVs, most notably the genera *Faecalibacterium* and *Roseburia* (Supplementary Table S1). No differences were observed in community richness or diversity, and none of the CD patients showed characteristics of severe gut community disruption, e.g., decreased diversity or overgrowth of facultative anaerobes from Enterobacteriales or *Enterococcus*.

### 3.2. GDMs Isolated from Feces and Saliva

Only a proportion of colonies grown on MCG3 plates had lysis zones and were further subcultured as GDMs (Table 1). In total, 45 strains were isolated: 12 from fecal samples and 33 from saliva samples. In total, 40 strains could be identified, belonging to 13 genera and 15 species. Most strains were bacterial, whereas eight were fungal and identified as *Candida albicans*. More strains were obtained with direct sample plating (*n* = 34) than after pre-enrichment (*n* = 11), and the obtained species differed between both protocols. Additionally, more strains were isolated under aerobic than under anaerobic conditions (37 vs. eight strains, respectively). Interestingly, more anaerobes were isolated from the saliva samples (*n* = 6) than from the fecal samples (*n* = 2).

Although the number of GDM-positive samples was similar between CD patients and HVs, the overall diversity of isolated GDMs was higher in HVs than in CD patients (Table 1). Additionally, saliva samples exhibited a higher diversity than fecal samples: 1–14 and 1–5 different isolates were isolated from a single GDM-positive saliva and fecal samples, respectively (Table 1). Three GDM species isolated from saliva were shared between CD patients and HVs (*C. albicans*, *R. mucilaginosa*, and *Streptococcus salivarius*) (Table 1). More GDMs were isolated from fecal samples from HVs than from CD patients, and there was no species overlap between the groups. Three species, *Klebsiella aerogenes*, *Veillonella atypica*, and *C. albicans*, were present in both saliva and feces. Among these, only *K. aerogenes* was isolated from the same individual (HV3) (Table 1).

### 3.3. The Detection of Operational Taxonomic Units (OTUs) after Different Cultivation Approaches of Feces and Saliva Samples from CD Patients and HVs

Microbial populations from directly inoculated plates, plates inoculated from enrichment broths, and enrichment broth cultures were subjected to 16S amplicon sequencing and OTU determination. While plates or enrichments incubated under aerobic or anaerobic conditions were sequenced separately (Supplementary Table S2), for further analysis, OTUs from anaerobic and aerobic conditions were merged for each individual and each cultivation condition (e.g., OTUs for CD1 were from directly cultivated saliva under aerobic and anaerobic conditions).

Altogether, 258 OTUs were detected from all cultivated samples, and 95 and 210 OTUs were determined from all obtained saliva and fecal cultures, respectively (Supplementary Table S2). *Lactobacillus*, *Streptococcus*, *Staphylococcus*, and *Veillonella* were most abundant in saliva cultures, and *Lactobacillus*, *Bifidobacterium* (OTU2 and OTU9), *Escherichia/Shigella*, *Enterococcus*, and *Bacteroides* were most abundant in fecal cultures.

**Figure 1:**
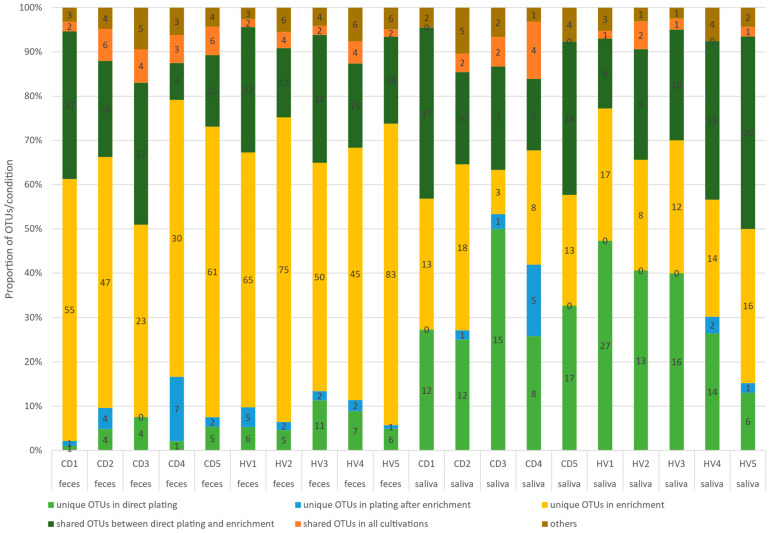
OTUs detected from different cultivation protocols. The term ‘others’ includes OTUs shared between direct plating or plating after enrichment and from enrichment and plating after enrichment. CD: celiac disease patient; HV: healthy volunteer.

We compared different cultivation approaches for each participant (Figure 1). For the saliva samples, enrichment and direct plating showed comparable numbers of unique OTUs. By contrast, for the fecal samples, enrichment yielded the highest number of unique OTUs, which were not detected by any other cultivation approach. As with the saliva samples, the overlap in OTUs between the enrichment broth and directly inoculated solid media was substantial (4–32 OTUs), and the diversity of populations on plates inoculated after enrichment was poor. The number of unique OTUs after direct plating was high (6–27) in saliva samples and lower (1–11) in fecal samples.

**Figure 2:**
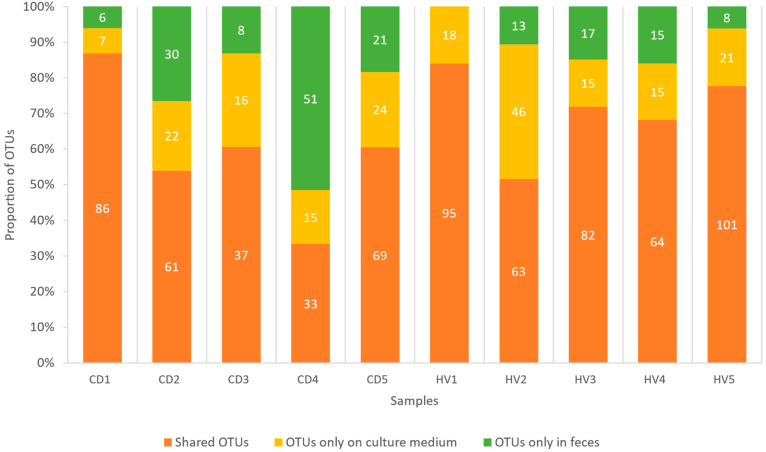
The number of operational taxonomic units (OTUs) detected in DNA isolated from feces and gluten-containing medium (merged results of different cultivation protocols; celiac disease (CD) patients, and healthy volunteers (HVs).

Furthermore, we also sequenced the original uncultured fecal sample. Next, we merged all the OTUs detected by any of the three different cultivation approaches and compared them with the original fecal sample (Figure 2). A substantial number of OTUs were detected in feces (45–92 in CD patients; 72–109 in HVs; Supplementary Table S2), and 33–101 OTUs were shared with cultivation-enriched fractions. Many OTUs (7–46) were only detected in fractions cultivated on gluten and not in the original uncultivated samples (Figure 2). The most represented genus that was only detected in cultivated samples was *Lactobacillus*, followed by *Enterococcus, Pseudomonas*, and two unidentified representatives from Enterobacteriaceae and Bifidobacteriaceae (Supplementary Table S2). The average percentage of OTUs not detected in feces but detected in gluten-containing medium did not differ between CD patients and HVs: 17.8% vs. 19.8%, respectively. Interestingly, the proportion of OTUs not detected by any of the cultivation approaches varied considerably among all ten individuals (0–51%).

### 3.4. SCFA Profiles of Fecal Samples from CD Patients and HVs

SCFA concentrations were measured for all 10 fecal samples. The total SCFA concentration was significantly higher (*p* = 0.047) in HVs than that in CD patients. Significantly lower acetic acid concentrations (*p* = 0.04) and a nonsignificant increase in caproic acid concentrations (*p* = 0.089) were observed in CD patients (Table 2). The average values of all other SCFAs were higher in HVs, but without any statistical significance.

SCFA concentrations were significantly correlated with the abundance of several bacterial groups (Supplementary Figure S1) but not with the overall bacterial diversity. Relative abundances of *Faecalibacterium* and *Actinomyces* were positively correlated with acetate concentration (Pearson’s correlation: *r* = 0.812, *p* = 0.004, and *r* = 0.781, *p* = 0.008, respectively). Community correlations with propionate and butyrate were less clear. The most significant positive correlations were between *Butyricimonas* and propionate (Pearson’s *r* = 0.768, *p* = 0.009) and a representative from Clostridiales and butyrate (Pearson’s *r* = 0.799, *p* = 0.006). Several significant correlations were also found for valerate concentrations, most notably positive correlations with *Coprococcus* (Pearson’s *r* = 0.837, *p* = 0.003) and *Dorea* (Pearson’s *r* = 0.769, *p* = 0.009) and a negative correlation with a representative from Clostridiales (Pearson’s *r* = 0.888, *p* < 0.001).

## 4. Discussion

GDMs are present in the environment and digestive tracts of animals and humans. They can potentially be used as probiotics or a source of gluten-degrading enzymes, which also exhibit a broad spectrum of applicability, from food supplements to gluten degradation for the production of gluten-free products [9]. Here, we compared culture-enriched saliva and fecal samples from adolescent HVs and CD patients with molecular profiling and isolated the GDMs in pure cultures. Gluten degradation was determined according to the presence of lysis zones on gluten medium; however, the exact degree of hydrolysis was not estimated, and its products were not further characterized.

More strains were obtained from HVs than from CD patients and from saliva than from feces. Most isolated strains were bacterial, and *C. albicans* was the only fungal representative. In a previous study, *C. albicans* was isolated from 67% of CD patients with gastrointestinal symptoms and was absent in healthy nonceliac controls [20]; however, its role in CD is unclear [21]. In our study, *C. albicans* with gluten-degrading properties was present in the saliva and feces of one CD patient and one HV.

The bacterial isolates in this study mostly belonged to known gluten-metabolizing genera such as *Rothia*, *Staphylococcus*, and *Lactobacillus*, as already described [9,18]. Most of them were isolated under aerobic conditions; however, three strict anaerobes were also detected. Of these, *Prevotella histicola* is known as a GDM, while *Veillonella* species (*V. atypica* and *V. parvula*) are not. As there is a diverse variety of gluten-degrading strains [9], our discovery of new microbiota representatives with GDM properties is not surprising. The ability to degrade gluten varies amongst representatives of the same species. Most bacteria found in our and other studies play known roles in microbiota–host interactions, such as immune modulation and maintenance of epithelial barrier function [22]. Some bacteria, e.g., *Klebsiella*, relocate from the oral cavity to the gut, in which they promote inflammatory processes [23]. In this study, we also isolated *K. aerogenes* from the saliva and feces of one of the participants.

Using different cultivation approaches for the molecular profiling of bacterial populations revealed differences. While there was some overlap in the OTUs detected by sequenced populations after direct plating, enrichment, and inoculating plates from the enrichment, each of these cultivated populations also had cultivation-specific OTUs. Direct plating and enrichment cultures of saliva and fecal samples showed considerable overlap. Interestingly, bacteria did not grow well on the solid media after cultivation in enrichment broth. This suggests that the enrichment step, as performed here, did not increase the isolation rate of GDM. However, enrichment or direct plating increased the overall detected diversity. A substantial proportion of OTUs detected by 16S amplicon sequencing in all the fractions cultivated on gluten medium were below the detection level in the original fecal sample. To better characterize the differences between celiac and healthy microbiota, the sequencing depth could be increased to enhance the detection level and, thus, increase the detected diversity; however, this would increase the costs of the analyses. As shown here, sequencing culture-enriched samples in addition to original samples could serve as an alternative, as previously described for other populations [24].

Comparing gut microbiomes and SCFA profiles was not the main aim of this study, as the sample number was low. As already known for CD, we observed differences in the total fecal bacterial microbiota (Supplementary Table S1). Total SCFA values were lower in CD patients. Regarding the SCFA profiles, only acetic acid significantly differed between our HVs and CD patients. The minor difference in total SCFA probably mirrors the fact that CD in the patients was well controlled. Decreases in SCFAs are mainly observed during active CD and improve after 1 year of a gluten-free diet [25].

## 5. Conclusions

Fecal bacterial communities in CD patients were not dysbiotic but differed from HVs in microbial composition. This finding is consistent with some previous studies. The proportion of the microorganisms cultivated on gluten-containing medium was below the detection limit during the sequencing of the original sample. We identified a variety of GDMs in the saliva and feces of adolescent HVs and CD patients. Most of the strains are already known GDMs and can be further used to characterize specific enzymes or as potential probiotics.

## Figures and Tables

**Table 1:** An overview of the isolated gluten-degrading microorganisms under different conditions.

	Feces		Saliva	
	Healthy Volunteers (HVs)	Celiac Disease (CD) Patients	Healthy Volunteers (HVs)	Celiac Disease (CD) Patients
No. of GDM-positive samples	3/5	2/5	3/5	3/5
No. of strains	9	3	21	12
Aerobic conditions	*Bacillus pumilus* (HV2, D) *Escherichia coli* (HV3, D) *Enterobacter cloacae* (HV2, D) *Klebsiella aerogenes* (HV3, D, 3 strains), *Lactobacillus paracasei* (HV4, E) *Lactobacillus plantarum* (HV4, E) *Paenibacillus pasadenensis* (HV3, E)	*Candida albicans* (CD3, E)	*Candida albicans* (HV4, D, 3 strains) *Rothia mucilaginosa* (HV1, D, 3 strains; HV4, D, 3 strains) *Streptococcus salivarius* (HV4, D) *Klebsiella aerogenes* (HV3, D) *Micrococcus luteus* (HV3, D, 2 strains) *Staphylococcus epidermidis* (HV1, D; HV4, D)	*Candida albicans* (CD3, E, 5 strains) *Rothia mucilaginosa* (CD1, D) *Streptococcus salivarius* (CD2, D) 5 unidentified strains (CD2, D) *
Anaerobic conditions		*Veillonella atypica* (CD1, E, 2 strains)	*Veillonella atypica* (HV4, D) *Veillonella parvula* (HV4, D) *Prevotella histicola* (HV4, D, 4 strains)	

D: direct cultivation without enrichment; E: cultivation with pre-enrichment step; GDM: gluten-degrading microorganism. * Five recovered isolates could not be identified by MALDI-TOF.

**Table 2:** Individual SCFA concentrations (µmol/g of feces) and total SCFA values in fecal samples from celiac disease (CD) patients and healthy volunteers (HV).

	Acetic Acid (Acetate)	Propionic Acid (Propionate)	Isobutyric Acid	Butyric Acid (Butyrate)	Isovaleric Acid	Valeric Acid (Valerate)	Caproic Acid	Total SCFA
*p*-Value	0.040	0.222	0.150	0.169	0.469	0.393	0.089	0.047
HV Average (SD)	124.987 (25.988)	19.838 (6.856)	3.099 (0.708)	13.740 (3.857)	4.042 (1.169)	2.304 (0.407)	0.143 (0.026)	168.154 (34.1)
CD Average (SD)	92.377 (14.348)	14.798 (5.056)	2.297 (0.877)	9.412 (5.084)	3.388 (1.528)	1.798 (1.185)	0.747 (0.698)	124.817 (20.125)

## Data Availability

Datasets are available from the authors upon request.

## Supplementary Materials

The following are available online at https://www.mdpi.com/article/10.3390/microorganisms9112375/s1. Figure S1: Correlations between SCFAs and relative abundances of different bacterial groups; Table S1: LefSe analysis of differentially represented bacterial groups between celiac disease patients and healthy controls; Table S2: Summary of OTUs and relative abundances detected with 16S amplicon sequencing of original fecal samples, culture-enriched fecal samples, and culture-enriched saliva samples (Excel file).
