# Supplementary material for: Comparison of Microbial Populations in Saliva and Feces from Healthy and Celiac Adolescents with Conventional and Molecular Approaches after Cultivation on Gluten-Containing Media: An Exploratory Study
Source: Microorganisms. 2021 Nov 17;9(11):2375. doi: 10.3390/microorganisms9112375 (PMC8623131; doi:10.3390/microorganisms9112375)
Supplement: Supplementary file 1 [file microorganisms-09-02375-s001.zip › Supplementary figure S1.pdf]

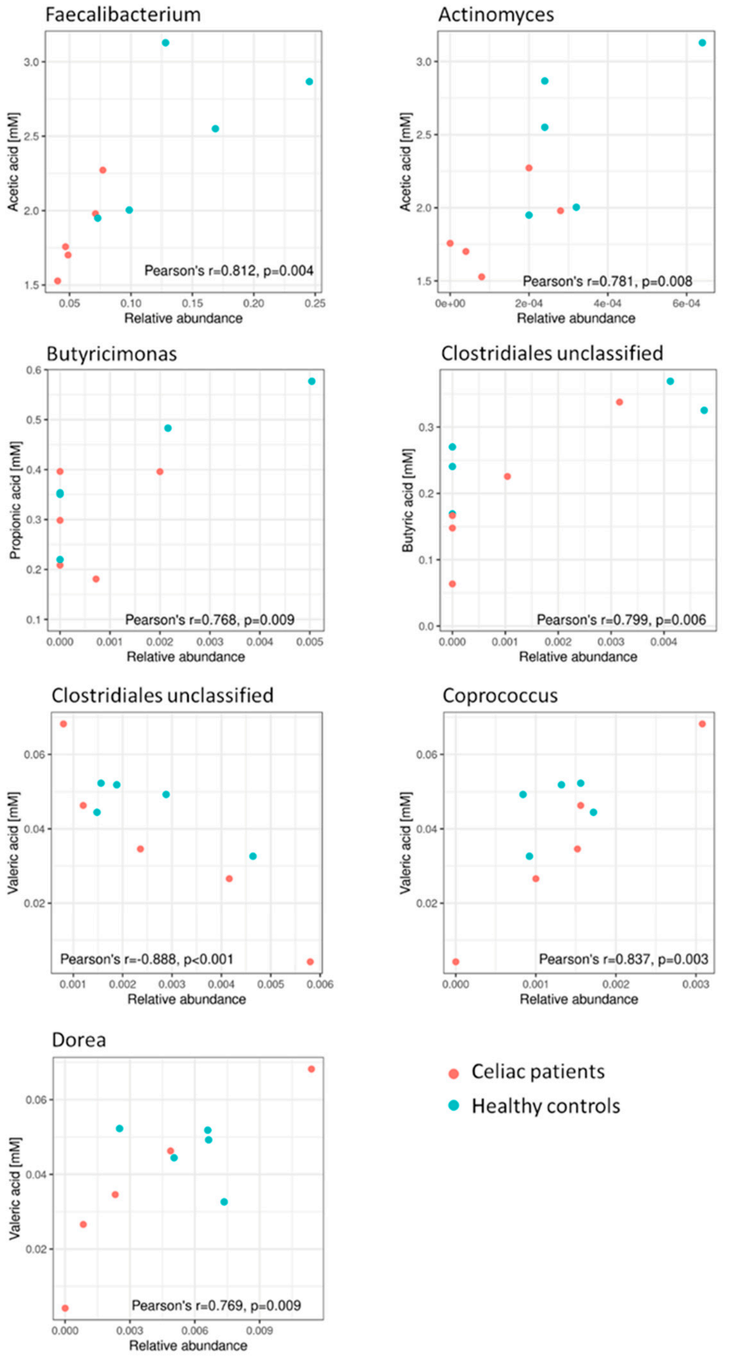

Supplementary figure S1. Correlations between SCFAs and relative abundances of different bacterial groups. Correlations were obtained with Pearson's correlation test. Figure presents most significant correlations, the criteria was p < 0.01.
