# Supplementary material for: Comparison of Microbial Populations in Saliva and Feces from Healthy and Celiac Adolescents with Conventional and Molecular Approaches after Cultivation on Gluten-Containing Media: An Exploratory Study
Source: Microorganisms. 2021 Nov 17;9(11):2375. doi: 10.3390/microorganisms9112375 (PMC8623131; doi:10.3390/microorganisms9112375)
Supplement: Supplementary file 1 [file microorganisms-09-02375-s001.zip › Supplementary Table S1.pdf]

Supplementary Table S1: LefSe analysis of differentially represented bacterial groups between celiac patients and healthy controls.

| **OTU** | **Increased in** | **LDA** | **pValue** |
|---|---|---|---|
| *Faecalibacterium* (OTU2) | Healthy controls | 4.63827 | 0.016302 |
| *Bacteroides* (OTU3) | Healthy controls | 4.06031 | 0.0472069 |
| *Roseburia* (OTU4) | Healthy controls | 4.04447 | 0.0445435 |
| *Fusicatenibacter* (OTU8) | Healthy controls | 3.65792 | 0.028295 |
| *Ruminococcaceae* (OTU24) | Healthy controls | 3.29666 | 0.0278177 |
| *Lachnospiraceae* (OTU38) | Healthy controls | 3.15978 | 0.028295 |
| *Ruminococcus* (OTU48) | Healthy controls | 3.07323 | 0.0264019 |
| *Butyricicoccus* (OTU70) | Healthy controls | 2.5224 | 0.028295 |
| *Ruminococcus* (OTU75) | Healthy controls | 3.48874 | 0.0186115 |
| *Eggerthella* (OTU91) | Healthy controls | 3.12828 | 0.0472069 |
| *Gordonibacter* (OTU243) | Healthy controls | 2.58244 | 0.0259355 |
| *Bacteroides* (OTU286) | Healthy controls | 3.05764 | 0.0186115 |
| *Blautia* (OTU45) | Celiac patients | 3.45325 | 0.00902436 |
| *Romboutsia* (OTU46) | Celiac patients | 3.29875 | 0.028295 |
| *Ruminococcaceae* (OTU249) | Celiac patients | 2.80513 | 0.0181607 |
